# Therapeutic efficacy of a K5-specific phage and depolymerase against *Klebsiella pneumoniae* in a mouse model of infection

**DOI:** 10.1186/s13567-024-01311-z

**Published:** 2024-05-07

**Authors:** Pei Li, Genglin Guo, Xiangkuan Zheng, Sixiang Xu, Yu Zhou, Xiayan Qin, Zimeng Hu, Yanfei Yu, Zhongming Tan, Jiale Ma, Long Chen, Wei Zhang

**Affiliations:** 1 MOE Joint International Research Laboratory of Animal Health and Food Safety, College of Veterinary Medicine, Nanjing Agricultural University, Key Lab of Animal Bacteriology, Ministry of Agriculture, Nanjing, 210095 China; 2 The Sanya Institute of Nanjing Agricultural University, Yabulun Industrial Park, Yazhou Bay Science and Technology City, Sanya, 572024 China; 3 Shandong Institute of Sericulture, Shandong Academy of Agricultural Sciences, Yantai, China; 4 Institute of Veterinary Medicine, Jiangsu Academy of Agricultural Sciences, Key Laboratory of Veterinary Biological Engineering and Technology, Ministry of Agriculture and Rural Affairs, Nanjing, China; 5 NHC Key Laboratory of Enteric Pathogenic Microbiology, Jiangsu Provincial Center for Disease Control and Prevention, Nanjing, 210014 China; 6 Department of Clinical Laboratory, Zhangjiagang Hospital Affiliated to Soochow University, Zhangjiagang, 215600 China

**Keywords:** *Klebsiella pneumoniae*, phage, depolymerase, K5, hypervirulent, capsule

## Abstract

*Klebsiella pneumoniae* has become one of the most intractable gram-negative pathogens infecting humans and animals due to its severe antibiotic resistance. Bacteriophages and protein products derived from them are receiving increasing amounts of attention as potential alternatives to antibiotics. In this study, we isolated and investigated the characteristics of a new lytic phage, P1011, which lyses K5 *K. pneumoniae* specifically among 26 serotypes. The K5-specific capsular polysaccharide-degrading depolymerase dep1011 was identified and expressed. By establishing murine infection models using bovine strain B16 (capable of supporting phage proliferation) and human strain KP181 (incapable of sustaining phage expansion), we explored the safety and efficacy of phage and dep1011 treatments against K5 *K. pneumoniae*. Phage P1011 resulted in a 60% survival rate of the mice challenged with *K. pneumoniae* supporting phage multiplication, concurrently lowering the bacterial burden in their blood, liver, and lungs. Unexpectedly, even when confronted with bacteria impervious to phage multiplication, phage therapy markedly decreased the number of viable organisms. The protective efficacy of the depolymerase was significantly better than that of the phage. The depolymerase achieved 100% survival in both treatment groups regardless of phage propagation compatibility. These findings indicated that P1011 and dep1011 might be used as potential antibacterial agents to control K5 *K. pneumoniae* infection.

## Introduction

*Klebsiella pneumoniae* is an important pathogenic bacterium that contributes to substantial economic loss in the dairy industry via mastitis infections and is also associated with many nosocomial infections [1, 2]. Although drug-resistant *K. pneumoniae* is not common in the dairy industry, restrictive policies for the use of antibiotics and withdrawal time make it difficult to combat these bacterial infections. Owing to the unreasonable use of antibiotics, *K. pneumoniae* has developed resistance to an array of antimicrobial agents in hospital environments [3, 4]. Moreover, carbapenem-resistant *K. pneumoniae* (CRKP) and polymyxin-resistant *K. pneumoniae* have surfaced worldwide, sparking global alarm [5–7]. *K. pneumoniae* was considered a critical-priority bacteria by the World Health Organization in 2017 [8].

*K. pneumoniae* is characterized by a thick capsule, and its virulence relies heavily on this feature [9]. The hyperspermucoviscous phenotype is related to excessive capsule production [10]*.* Strains accompanied by the myxoid type are usually deemed hypervirulent *K. pneumoniae* (hvKP) [11]. The K1, K2, K5, K20, K54 and K57 serotypes are strongly linked with hvKP [12]. In addition, excessive capsule production contributes to biofilm formation, which generates obvious effects on the efficacy of antibiotics. Hence, innovations in *K. pneumoniae* management strategies beyond conventional antibiotics are urgently needed.

Phage therapy has resurfaced as a potentially effective alternative because of increasing antibiotic resistance. Phages are viruses that can infect and eliminate specific bacteria. Some reports have proven the effectiveness of antibacterial phages against *Klebsiella* [13, 14]*.* However, most studies on *Klebsiella* phages have focused predominantly on the K1, K2 and KL64 serotypes [15, 16]. The evaluation of phage antibacterial properties against K5 *K. pneumoniae* remains scarce and limited to only one existing assessment [17]. Here, we successfully isolated and characterized a new K5-specific phage bearing negligible similarity to prior K5 *Klebsiella* phages, bolstering the armamentarium of phages available for controlling K5 *K. pneumoniae* infections.

In addition to intact phages, phage-derived proteins, such as depolymerases and lysin, also exhibit promising prospects for combating diverse bacterial pathogens [18, 19]. To date, more than two dozen *K. pneumoniae*-targeting depolymerases have been identified, including diverse capsular serotypes such as K1, K2, K3, K5, K8, K11, K13, K19, K21, K23, K25, K30, K35, K47, K51, K56, K57, K63, K64, and K69, as well as new serotypes KN1, KN2, KN3, KN4 and KN5 [20]. Notably, despite the discovery of two K5-specific depolymerases, there have been no relevant reports on their antimicrobial potential [17].

Here, we established a mouse infection model with both a bovine strain (supporting phage proliferation) and a human strain (not supporting phage proliferation) of *K. pneumoniae* simultaneously. The therapeutic efficacy of K5 *Klebsiella* phage and depolymerase against two different bacteria in a mouse model was evaluated for the first time.

## Materials and methods

### Bacterial strains and culture

A total of 38 K*. pneumoniae* strains were used to assess the host range. All strains were cultivated in Luria–Bertani broth and a string test was performed. The serotype of the strains was identified by Kaptive 2.0 and polymerase chain reaction [21, 22]. The primers used in this study were listed in Table 1.

**Table 1:** Primers used in this study.

Primers	Sequence (5’ to 3’)
K1F	GGTGCTCTTTACATCATTGC
K1R	GCAATGGCCATTTGCGTTAG
K2F	GACCCGATATTCATACTTGACAGAG
K2R	CCTGAAGTAAAATCGTAAATAGATGGC
K5F	TGGTAGTGATGCTCGCGA
K5R	CCTGAACCCACCCCAATC
K20F	CGGTGCTACAGTGCATCATT
K20R	GTTATACGATGCTCAGTCGC
K54F	CATTAGCTCAGTGGTTGGCT
K54R	GCTTGACAAACACCATAGCAG
K57F	CTCAGGGCTAGAAGTGTCAT
K57R	CACTAACCCAGAAAGTCGAG
*bla*_KPC_F	GTATCGCCGTCTAGTTCTGC
*bla*_KPC_R	GGTCGTGTTTCCCTTTAGCC

### Conjugation and carbapenem resistance

Conjugation was performed as previously described with minor changes [23]. To evaluate the ability of plasmids to mobilize carbapenem-resistant bacteria, strains B16 and KP181 were used as recipient bacteria, and KP492 was used as the donor strain. These strains were cultivated to an OD_600_ of 0.5 using Mueller–Hinton (MH) medium. Then, 50 µL of KP492 was mixed with 150 µL of the recipient bacteria and the mixture was incubated at 28 ℃ for 20 h. The bacteria were grown on MHA plates containing chloramphenicol, spectinomycin and meropenem and then identified by primers for the *bla*_KPC_ and K5 serotypes.

Carbapenemase from the positive bacteria was identified by a modified Hodge test as previously described [24]. A bacterial suspension of ATCC25922 at 0.5 MCF was diluted with MH at 1:10. The suspension was spread on the MHA plate equally, and a 10 µg meropenem disk was placed on the dried plate. Five positive colonies on the blood plate were picked up by an inoculation loop, and a line was drawn from the edge to the middle of the plate.

The minimum inhibitory concentration (MIC) of meropenem was measured using a Phoenix NMIC-413 AST panel following the Clinical and Laboratory Standards Institute (CLSI) standards.

### Phage isolation and morphology

Wastewater from the Second Affiliated Hospital of Soochow University was used for phage isolation. After centrifugation and filtration using a 0.22 μm filter membrane, 200 μL of sample was mixed with 100 μL *K. pneumoniae* B16 for phage isolation. The double-layer plating method was used to isolate, purify and propagate the phage. The morphological characteristics of the phage were observed by transmission electron microscopy (H-7650; Hitachi, Tokyo, Japan) at 100 kV.

### Phage host range and characteristics

Spotting tests and double-layer plating methods were used for evaluating the host range as previously described [25]. Specifically, 100 μL of phage (1.0 × 10^9^ PFU/mL) was dropped on a lawn plate containing *K. pneumoniae*. Then, 100 μL of phage (1.0 × 10^9^ PFU/mL) mixed with *K. pneumoniae* was added for determination of the lysis spectrum.

The stability was evaluated as previously described [26]. One millilitre of phage (1.0 × 10^9^ PFU/mL) was added to a thermostatic water bath at 4, 25, 37, 50 or 60 °C for 1 h. Then, 100 μL of phage (1.0 × 10^9^ PFU/mL) was mixed with 900 µL of SM buffer at pH 3–12 to evaluate the pH stability for 1 h.

Tenfold dilutions of phage were serially cocultivated with 6 mL of *K. pneumoniae* B16 (3.0 × 10^8^ CFU/mL) at various multiplicities of infection (MOIs) (PFU/CFU = 10, 1, 0.1, 0.01, 0.001 and 0.0001) for 10 h [26]. A one-step growth assay was then performed to measure the latency and burst size at an MOI of 0.1 [26]. The phage titre was calculated by the number of plaques. The above assay was repeated in triplicate.

### Phage genome extraction and annotation

The genomic DNA of 8 mL of phage (1.0 × 10^9^ PFU/mL) was extracted using a λ Phage Genomic DNA Extraction Kit (Cat #AB1141, Abigen, China). The phage genome was sequenced via an Illumina HiSeq system (Illumina, San Diego, CA, USA). Sequencing reads were de novo assembled through Spades 3.11.1. Phage lifestyle was identified by way of PhageScope [27]. RAST was used to predict putative coding sequences (CDSs) [28]. The functions of the predicted CDSs were analysed using Protein BLAST. The acquired antibiotic-resistance genes were screened using ResFinder [29]. Virulence factors were detected through the VFDB database [30]. The tRNA genes were searched by tRNAscan-SE [31].

### Phage genome comparison and phylogenetic analysis

Closely related phages were identified through online BLASTn. Ten closely related phage genomes were aligned for classification by Victor using Formula D0 [32]. A proteomic tree was generated through VIPTree based on phage genomes within one family [33]. By virtue of the amino acid sequences of the large terminase subunit, the evolutionary relationship of P1011 with 18 phages was determined using MEGA X. The amino acid sequence of the putative depolymerase was aligned with that of the K5-specific depolymerase previously reported using EMBL-EBI.

### Depolymerase cloning expression and activity

The predicted depolymerase gene was amplified using the primers ATGGGTCGCGGATCCGAATTCATGGCAATATACAGAGAAGGCAAA and CTCGAGTGCGGCCGCAAGCTTTTAGATCATGCAACCGTAACGC. The purified fragment was inserted into pET-28a through the EcoRI and HindIII sites. Recombinant depolymerase was expressed in BL21 (DE3) cells after induction with 0.5 mM isopropyl-beta-D-thiogalactopyranoside at 16 °C for 12 h. The His-tagged soluble protein was purified via a Ni-chelating chromatography column using 500 mM imidazole. Then, the purified protein buffer was replaced with PBS and concentrated using a 30 kDa ultrafiltration tube. The concentration of recombinant depolymerase was assessed by a BCA Protein Assay Kit (Thermo Scientific, USA).

To assess the enzymatic spectrum of depolymerase, 50 ng of depolymerase was dropped on the lawn of bacteria, and PBS was used as a negative control*.* Twenty millilitres each of strains B16 and KP181 at the logarithmic phase was centrifuged at 5000 *g* for 5 min and washed with PBS three times. The sediment was resuspended in 1 mL of PBS buffer or 1 mL of dep1011 and shocked for 6 h at 37 °C. Bacteria were collected at 5000 *g* for 5 min to observe any morphological differences.

### Mouse infection and treatment

To evaluate the curative effect of the phage and depolymerase in vivo, 9 groups (10 mice in Groups 1–6 and 5 mice in Groups 7–9) of 5-week-old female BALB/c mice were purchased from the Laboratory Animal Center of Yangzhou University. As described in a previous study, strain B16 was injected into mice at a dose of 1 × 10^8^ CFU in a volume of 200 μL, and strain KP181 was injected into mice at a dose of 4 × 10^7^ CFU in a volume of 200 μL [22]. A total of 200 μL of phage at a dose of 1 × 10^9^ PFU was intraperitoneally injected into each mouse in the phage treatment group, and 50 μg of isometric depolymerase was intraperitoneally injected into each mouse in the depolymerase treatment group.

The groups used were as follows: Group 1, positive control (mice infected with strain B16 and injected with 200 μL of PBS buffer 2 h after infection); Group 2, phage treatment (mice infected with strain B16 and phage injected 2 h after infection); Group 3, depolymerase treatment (mice infected with strain B16 and depolymerase injected 2 h after infection); Group 4, positive control (mice infected with strain KP181 and injected with 200 μL of PBS buffer injected 2 h after infection); Group 5, phage treatment (mice infected with strain KP181 and phage injected 2 h after infection); Group 6, depolymerase treatment (mice infected with strain KP181 and depolymerase injected 2 h after infection); Group 7, phage injected; and Group 8, depolymerase injected. Group 9 was injected with 200 μL of PBS as a control. All groups were observed for 7 days before euthanasia.

Sixteen hours after infection, the mice (5 mice in each infected group) were euthanized by cervical dislocation. The observed indices included viable bacteria in the blood, lung and liver. Blood samples were isolated from the eye veins of the mice. Viable bacteria in the blood, homogenized lung and liver were counted by plating serial dilutions. In addition, to observe histopathological changes, lung and liver tissues were fixed in 4% paraformaldehyde. Tissues were dehydrated, embedded in paraffin and stained with haematoxylin and eosin (H&E).

### Statistical analysis

All of the statistical data were analysed using GraphPad Prism 8. A mouse survival curve was generated using the log-rank (Mantel–Cox) test. A *P* value < 0.05 was considered to indicate statistical significance.

## Results

### Morphology and carbapenem resistance of bacteria

**Figure 1:**
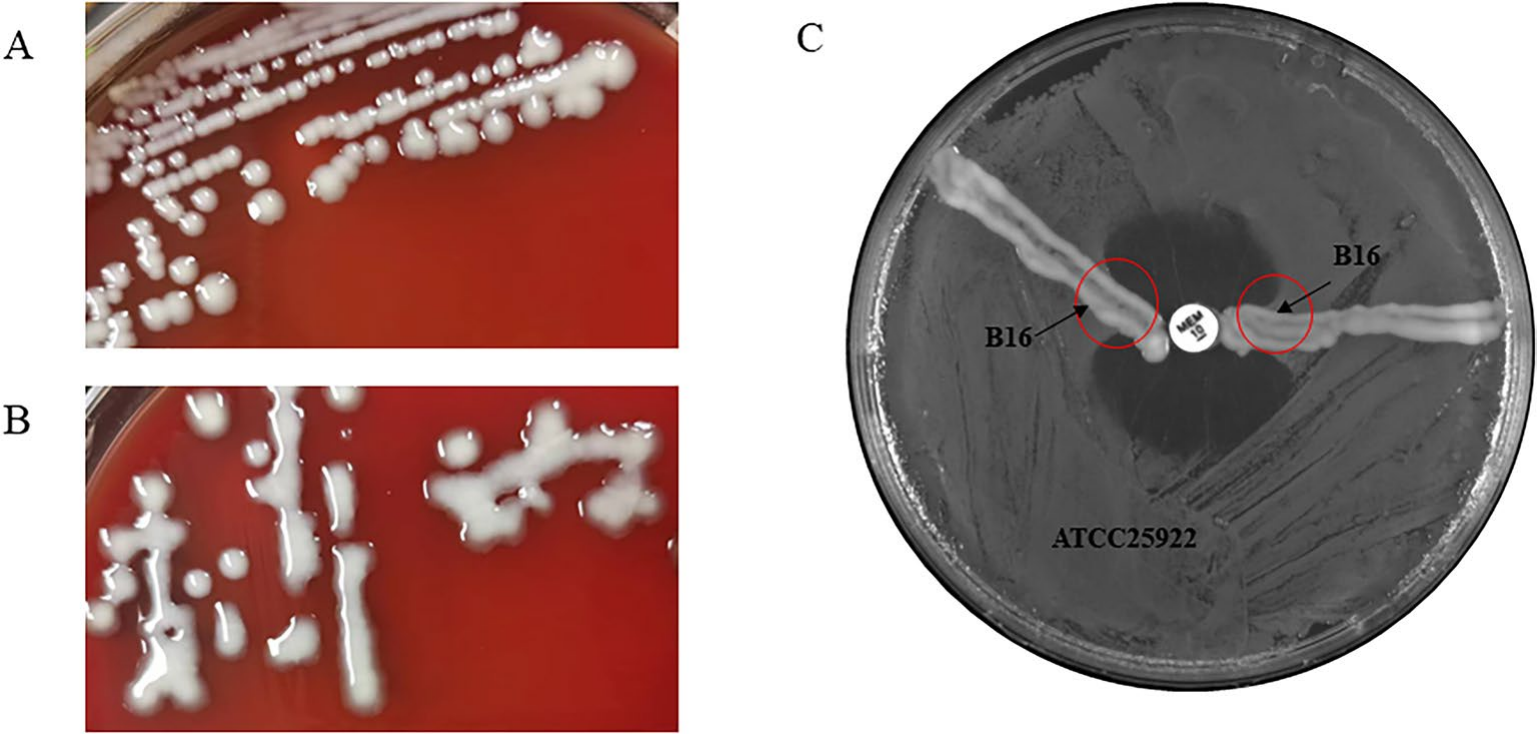
Morphology of strains. **A** Colony morphology of strain B16. **B** Colony morphology of strain KP181. **C** The results of the modified Hodge test for B16. KPC-2 was acquired from B16 through plasmid conjugation. As shown by the red circle, B16 hydrolysed meropenem, resulting in the growth of the strain ATCC25922 as an apple-like indentation in the inhibition zone.

Information on the strains used in this study is listed in Table 2. Strain B16 was negative according to the string test, and strain KP181 was positive according to the string test (Figures 1A, B). B16 successfully acquired the carbapenem resistance gene through plasmid conjugation, while KP 181 failed. The modified Hodge test indicated that B16 produced carbapenemase (Figure 1C). After the KPC-2 gene was amplified, the minimum inhibitory concentration of meropenem in strain B16 substantially increased (Table 3).

**Table 2:** Host range of phage P1011 and its enzymatic spectrum.

Bacteria	Year	Source	Collection site	KL/K	String test	Double plate	Spotting test	dep1011
KP1	2017	Human	Jiang su	KL25	**–**	**–**	**–**	**–**
KP3	2017	Human	Jiang su	KL14	**–**	**–**	**–**	**–**
KP29	2017	Human	Jiang su	KL139	**–**	**–**	**–**	**–**
KP72	2018	Human	Jiang su	K3	**–**	**–**	**–**	**–**
KP101	2018	Cow	Jiang su	K54	**–**	**–**	**–**	**–**
KP167	2022	Pig	Jiang su	K5	**–**	+	+	◎
B16	2018	Cow	Jiang su	K5	**–**	+	+	◎
KP181	2019	Human	Jiang su	K5	+	**–**	◎	◎
KP191	2020	Human	Jiang su	K1	+	**–**	**–**	**–**
KP216	2020	Cow	Shanghai	K57	**–**	**–**	**–**	**–**
KP218	2020	Human	Jiang su	K2	+	**–**	**–**	**–**
KP232	2021	Cow	Shanghai	KL30	**–**	**–**	**–**	**–**
KP248	2021	Cow	Shanghai	KL149	**–**	**–**	**–**	**–**
KP253	2021	Cow	Shanghai	KL103	**–**	**–**	**–**	**–**
KP313	2021	Cow	Shanghai	KL61	**–**	**–**	**–**	**–**
KP318	2021	Cow	Shanghai	KL21	**–**	**–**	**–**	**–**
KP341	2021	Human	Jiang su	KL112	**–**	**–**	**–**	**–**
KP345	2021	Human	Jiang su	KL110	**–**	**–**	**–**	**–**
KP365	2020	Human	Jiang su	KL24	**–**	**–**	**–**	**–**
KP370	2021	Human	Jiang su	KL116	**–**	**–**	**–**	**–**
KP435	2022	Human	Jiang su	K5	+	**–**	◎	◎
KP446	2022	Human	Jiang su	KL64	**–**	**–**	**–**	**–**
KP448	2022	Human	Jiang su	KL123	**–**	**–**	**–**	**–**
KP449	2022	Human	Jiang su	KL47	**–**	**–**	**–**	**–**
KP451	2022	Human	Jiang su	KL57	**–**	**–**	**–**	**–**
KP454	2022	Human	Jiang su	KL19	**–**	**–**	**–**	**–**
KP461	2022	Human	Jiang su	KL8	**–**	**–**	**–**	**–**
KP484	2022	Human	Jiang su	KL107	**–**	**–**	**–**	**–**
KP485	2022	Human	Jiang su	KL15	**–**	**–**	**–**	**–**
KP858	2021	Human	Jiang su	K5	+	**–**	◎	◎
KP861	2021	Human	Jiang su	K5	+	**–**	◎	◎
KP905	2021	Human	Jiang su	K5	+	**–**	◎	◎
56,414	2023	Human	Jiang su	K5	+	**–**	◎	◎
56,505	2023	Human	Jiang su	K5	+	**–**	◎	◎
56,516	2023	Human	Jiang su	K5	+	**–**	◎	◎
56,563	2023	Human	Jiang su	K5	+	+	+	◎
56,650	2023	Human	Jiang su	K5	**–**	+	+	◎
57,530	2023	Human	Jiang su	K5	+	+	+	◎

+ , clear plaques surrounded by a turbid halo; –, no plaques; ◎ turbid halo.

**Table 3:** MICs of meropenem for the strains.

Strain	MIC (μg/mL)
B16	< 0.125
B16 acquired KPC-2 gene	> 8

### Phage morphology

**Figure 2:**
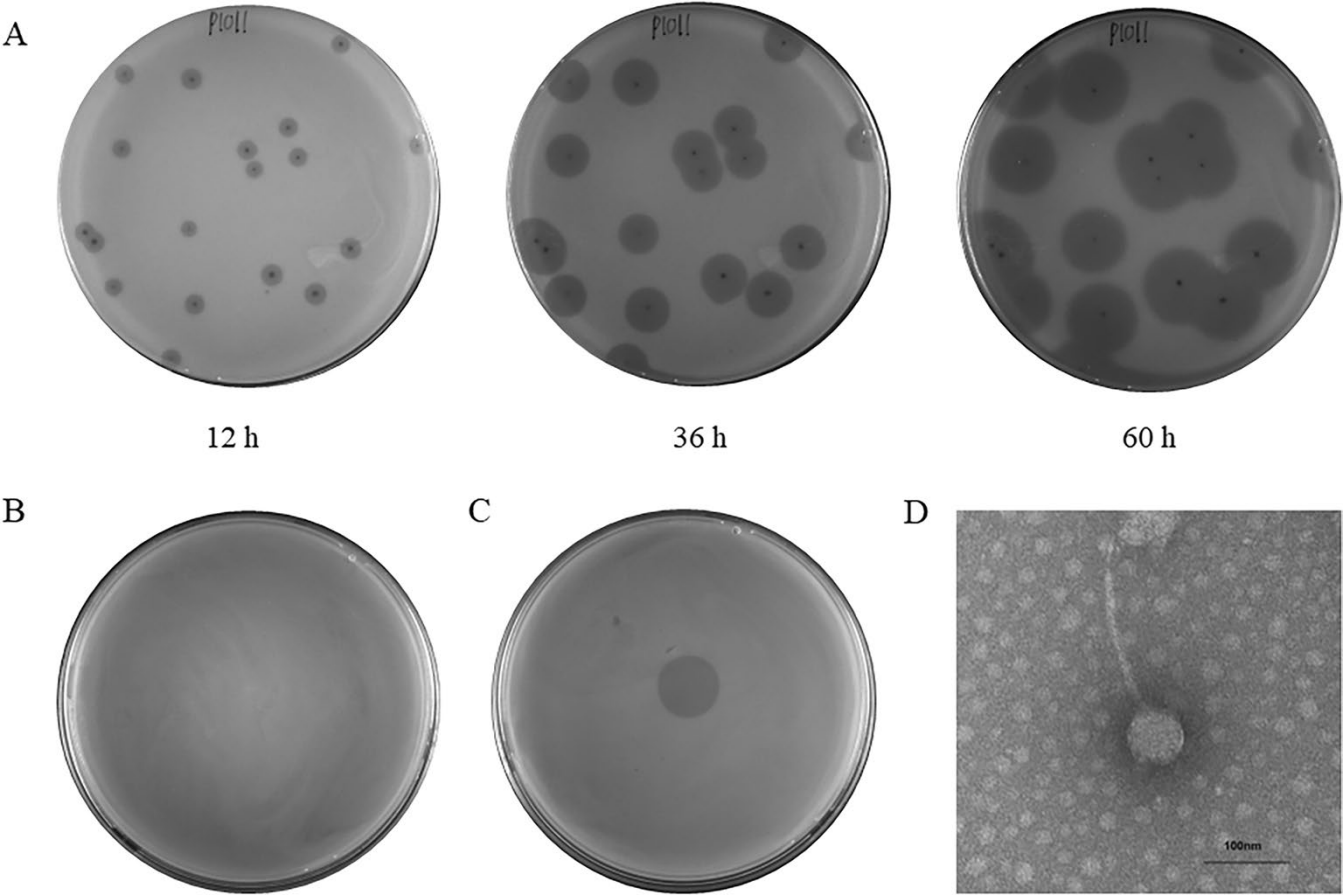
Morphology of the phage. **A** Plaque of P1011 using strain B16 as a host. P1011 plaques were observed at 12 h, 36 h and 60 h. As time progressed, the halo gradually became larger. **B** No plaques were generated by the double plate method using KP181 as the host. **C** The halo of P1011 formed on the lawn of KP181 according to the spotting test. **D** Transmission electron micrograph of phage P1011. The scale bar is 100 nm.

Phage P1011 could form small, round plaques surrounded by an aureole when using strain B16 as a host. Over time, the expansile aureole revealed that P1011 was likely to encode a depolymerase (Figure 2A). When KP181 was used as the host strain, although no plaques formed according to the double-plate method (Figure 2B), there was a turbid ring according to the spotting test (Figure 2C). P1011 possessed an isometric polyhedral head (65 ± 0.5 nm) and a long tail (168 ± 0.5 nm) (Figure 2D).

### Phage host range

Among the 26 serotypes, all strains lysed by phage P1011 belonged to the K5 serotype. Notably, not all strains within the K5 serotypes could be lysed. P1011 only formed a turbid halo on the lawn of part of the K5 strain serotype (Table 2).

### Phage growth characteristics and stability

**Figure 3:**
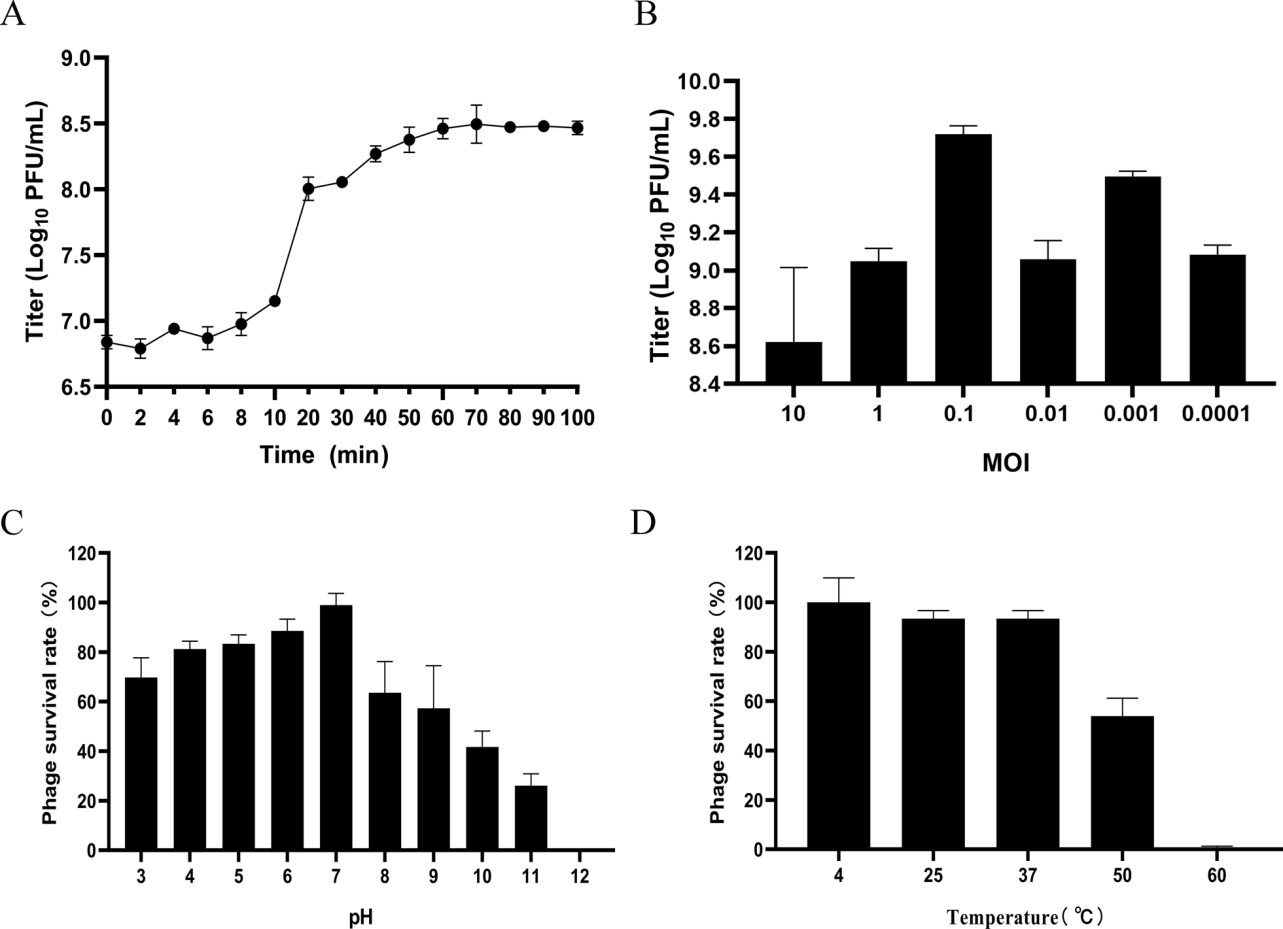
Biological characteristics of phages. **A** One-step growth curve of P1011. The sample was collected at certain intervals (every 2 min from 0 to 10 min and every 10 min from 10 to 100 min). **B** The optimal multiplicity of infection (MOI) of P1011 was evaluated. **C** pH stability of P1011. **D** Thermal stability of P1011.

According to the one-step growth curve, P1011 had an incubation period of 6 min and a burst size of approximately 41 virus particles per cell (Figure 3A). After 10 h of coculture, P1011 had the greatest number of offspring at an MOI of 10^–3^, revealing that 10^–3^ was the optimal MOI (Figure 3B). More than half of the P1011 strains survived at pH 3–9 (Figure 3C). The viability of P1011 under acidic conditions was greater than that under alkaline conditions. Over 90% of the phage particles remained active at 4, 25 and 37 ℃. With increasing temperature, the survival rate of P1011 rapidly decreased. More than 50% of the phage was viable at 50 ℃, but almost no phage survived at 60 ℃ (Figure 3D).

### Phage genome annotation and comparison

**Figure 4:**
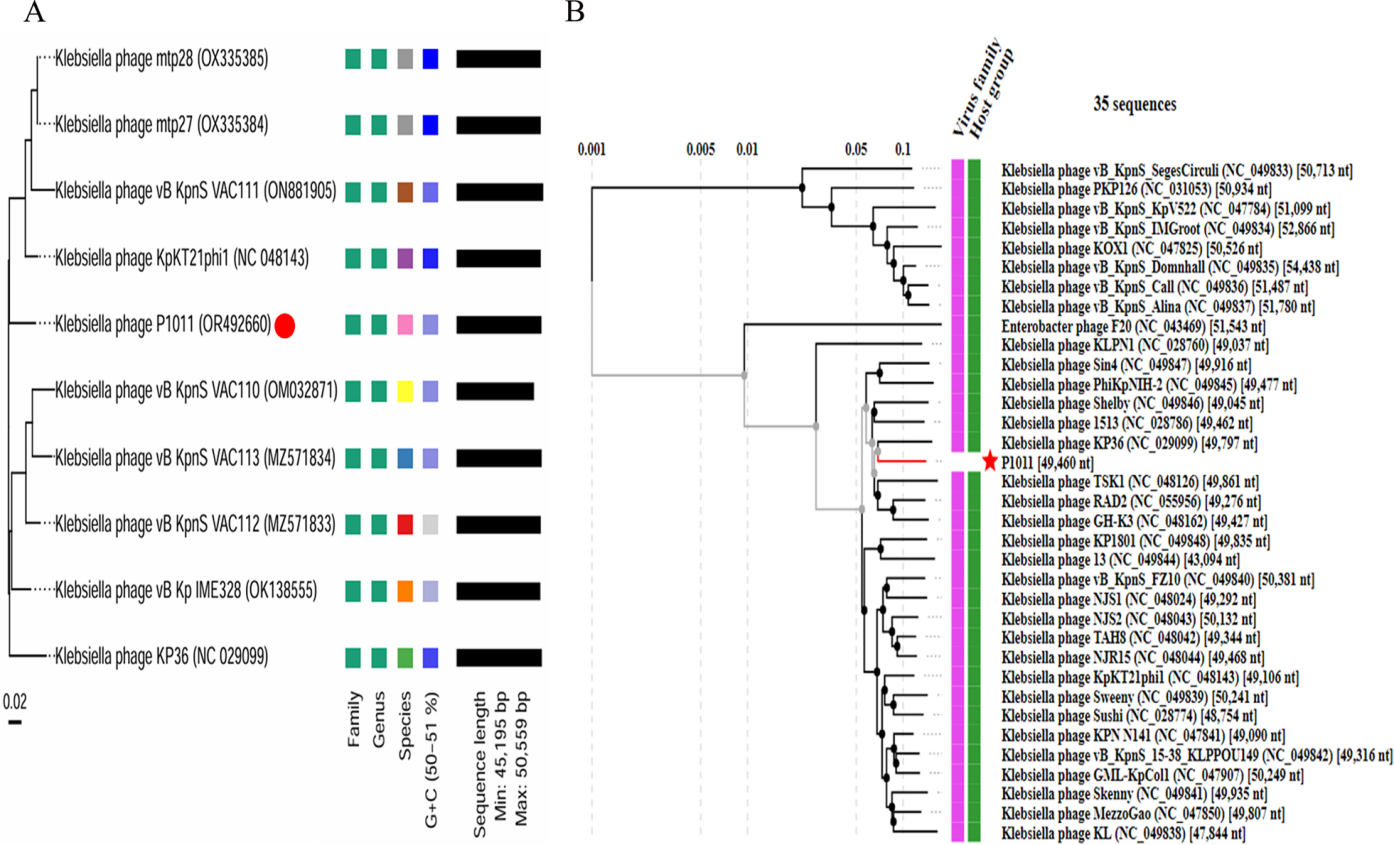
Phage genome comparison. **A** Whole-genome comparison of ten phages highly homologous to P1011. The comparison was performed through the Genome-BLAST Distance Phylogeny (GBDP) method by VICTOR using Formula D0. Phage P1011 is marked with a red circle. **B** Viral proteomic trees of phages. A viral proteomic tree of P1011 was constructed with 34 phages (those with greater than 50% genomic similarity). P1011 is marked with a red pentacle.

**Figure 5:**
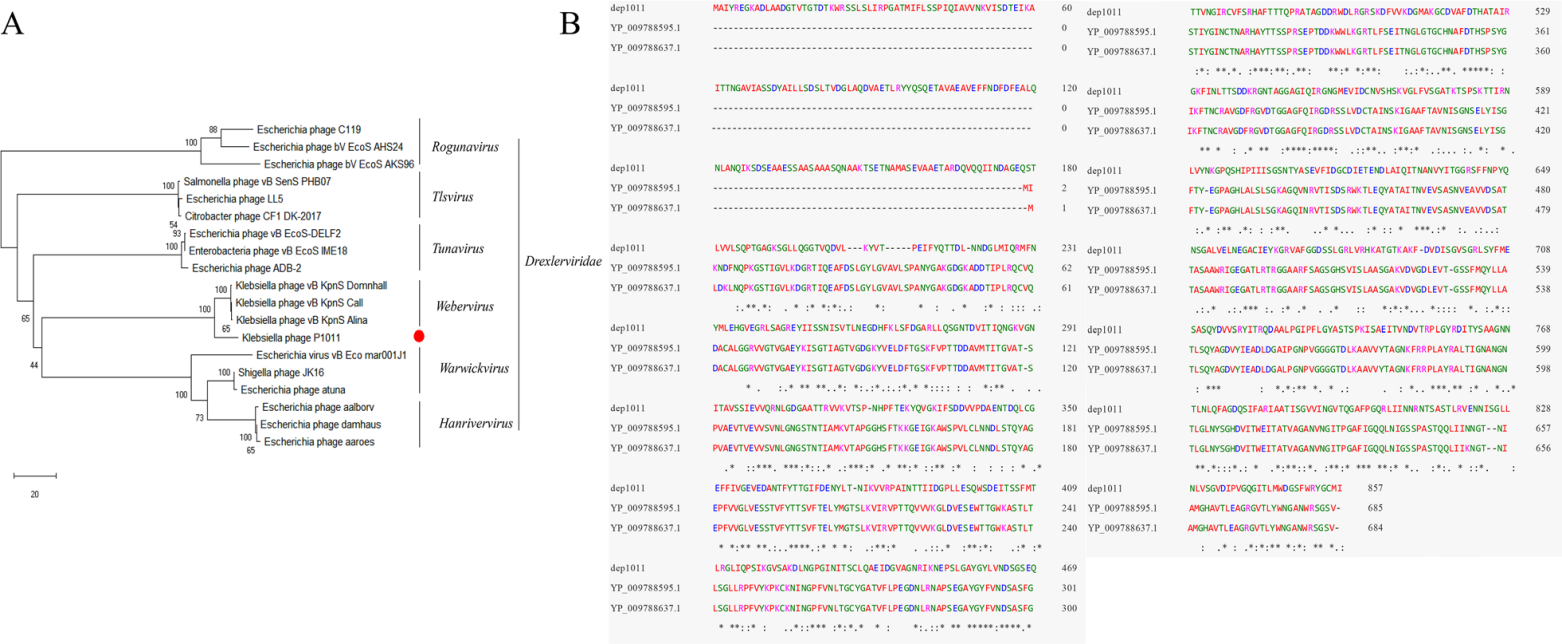
Protein phylogenetic analysis and comparison. **A** Phylogenetic analysis of the large terminase subunit. The phylogenetic analysis was conducted using the neighbour-joining method with 1000 bootstrap replicates in MEGA X. **B** Protein alignment of K5 depolymerase. Amino acid sequence alignment of the putative depolymerase P1011 with a previously reported K5 depolymerase.

The P1011 genome was deposited in GenBank under accession number OR492660. The genome was composed of 49 460 bp with 50.41% GC content. There were no lysogenic, antibiotic-resistant, virulence factor-related or tRNA-relevant genes detected in the genome. BLASTn showed that P1011 had high homology to *Klebsiella* phage vB_KpnS-VAC112 (accession number: MZ571833), with 88% query coverage and 94.24% nucleotide identity, and *Klebsiella* phage vB_KpnS-VAC111 (accession number: ON881905), with 88% query coverage and 94.33% nucleotide identity. Victor comparison using Formula D0 showed that P1011 belonged to the same genus as the ten phages (Figure 4A). VIPTree analysis revealed that P1011 and *Klebsiella* phage KP36 were derived from the same root and generated a detached clade (Figure 4B). A phylogenetic tree of the amino acid sequence of the large terminase subunit revealed a close relationship with phages within the genus *Webervirus* (Figure 5A)*.* Thus, P1011 should be a new species in the genus *Webervirus*, family *Drexlerviridae*, order *Caudoviricetes*. A total of 79 CDSs, including those of endolysin, holin and two-tail fibre proteins, were predicted. The amino acid sequence of CDS34 had partial homology to K5 depolymerase (accession number: YP_009788637) of *Klebsiella* phage K5-4 with 78% query coverage and 34.06% sequence identity and K5 depolymerase (accession number: YP_009788595) of *Klebsiella* phage K5-2 with 71% query coverage and 34.94% sequence identity, revealing that it may have enzymatic activity (Figure 5B).

### Depolymerase expression and activity

**Figure 6:**
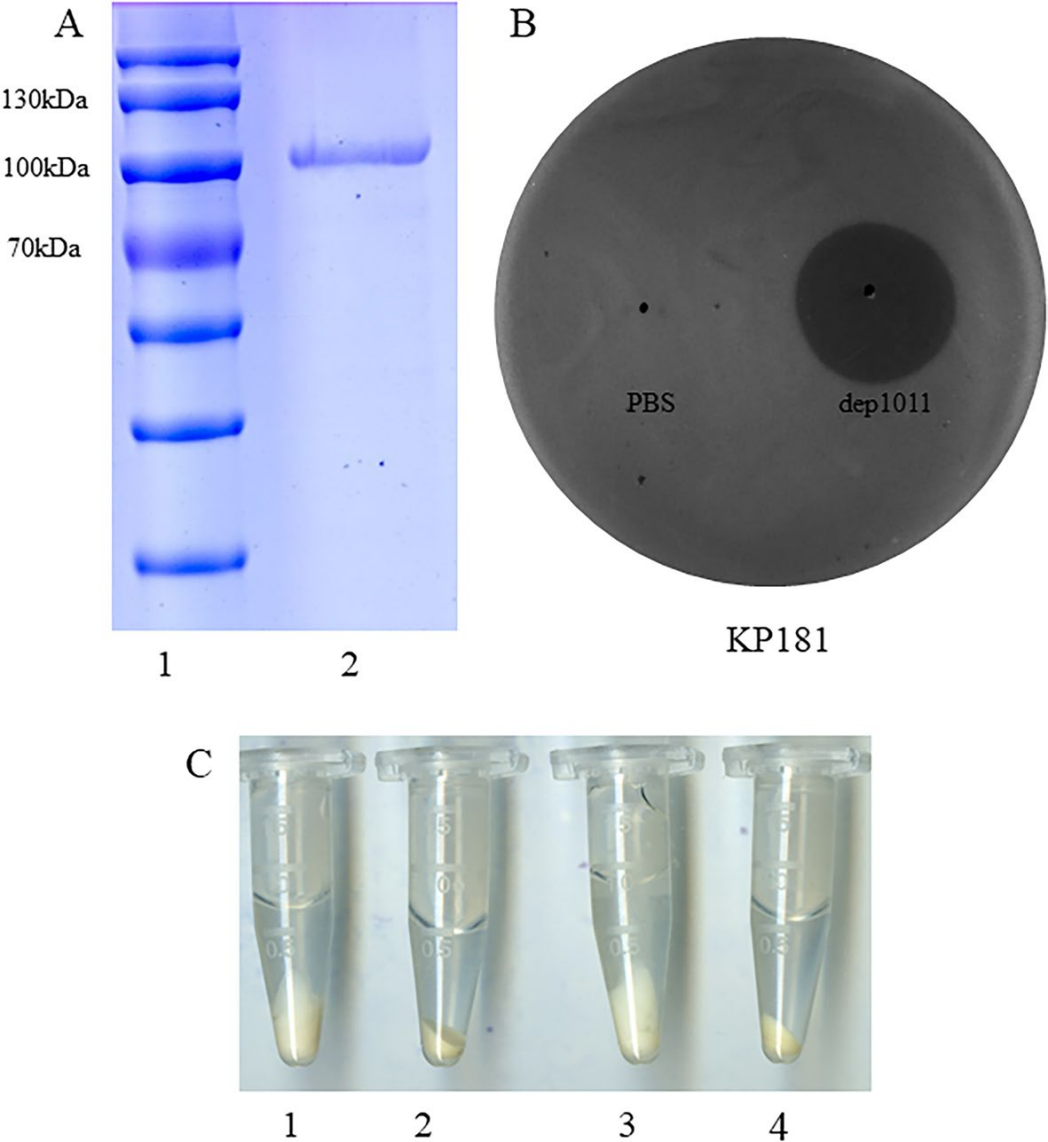
Protein expression and activity. **A** SDS‒PAGE of recombinant depolymerase. Lane 1, protein marker; Lane 2, purified depolymerase dep1011. **B** Depolymerase activity against KP181. First, 50 ng of dep1011 or PBS was spotted on the lawn of KP181. **C** Bacterial sediment of wild-type strains and strains treated with depolymerase. 1, Bacterial sediment of B16; 2, bacterial sediment of B16 treated with depolymerase; 3, bacterial sediment of KP181; 4, bacterial sediment of KP181 treated with depolymerase.

The recombinant depolymerase named dep1011 was successfully induced and purified. The predicted molecular weight of dep1011 was 92 kDa, and dep1011 presented as a single band at approximately 100 kDa on a 10% SDS‒PAGE gel (Figure 6A). A spotting test indicated that 50 ng of dep1011 could form a turbid halo on all K5 strains, including those strains that could not be lysed by P1011 (Figure 6B and Table 2). Following exposure to dep1011, the precipitates of both bacterial strains B16 and KP181 demonstrated greater compaction than those subjected to PBS (Figure 6C).

### Evaluation of phage and depolymerase protection

**Figure 7:**
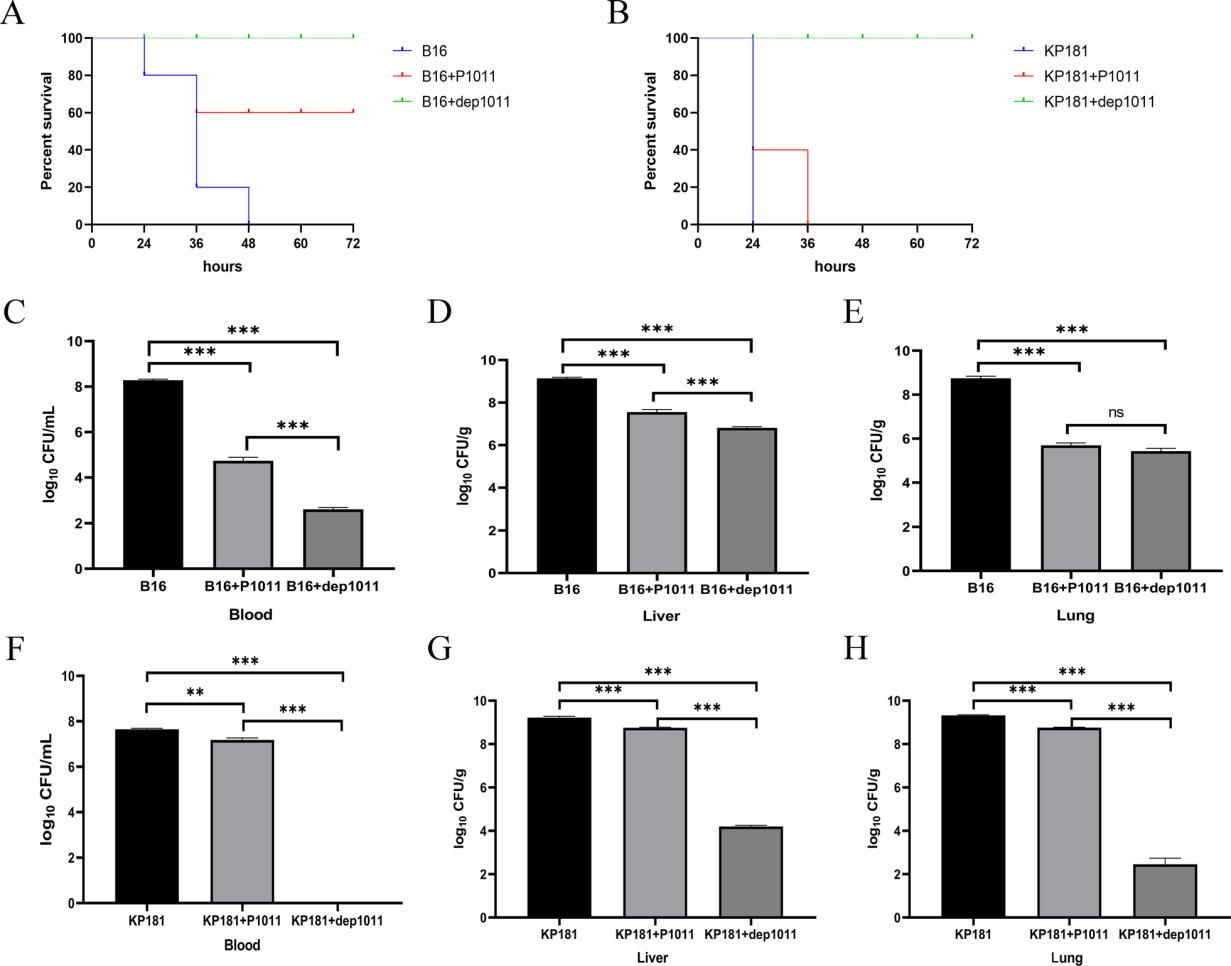
Therapeutic efficacy of phage and depolymerase. **A** Survival of mice infected with B16 and treated with P1011 and dep1011. **B** Survival of mice infected with KP181 and treated with P1011 and dep1011. **C** Viable bacteria in the blood of mice infected with B16 and in the treatment group. **D** Viable bacteria in the liver of mice infected with B16 and in the treatment group. **E** Viable bacteria in the lungs of mice infected with B16 and in the treatment group. **F** Viable bacteria in the blood of mice infected with KP181 and in the treatment group. **G** Viable bacteria in the liver of mice infected with KP181 and in the treatment group. **H** Viable bacteria in the lungs of mice infected with KP181 and in the treatment group.

Mice infected with strain B16 (supporting phage multiplication) all died within 48 h without intervention. Sixty percent of the mice in the phage treatment group survived, and all of the mice in the depolymerase treatment group survived (Figure 7A). Moreover, the bacterial counts in the blood, lungs, and livers of the mice treated with either agent were significantly lower than those in the control group (Figures 7C–E). In these two treatment groups, statistically significant differences emerged in the bacterial loads found in blood and liver samples but not in those collected from the lungs.

Mice infected with strain KP181 (not supporting phage multiplication) all died in the absence of therapeutic interventions in Group 4 within 24 h (Figure 7B). Interestingly, although all of the mice in Group 5 died, the bacterial burdens in their blood, lungs, and liver were still markedly diminished relative to those in the control cohort (Figure 7F–H). All of the mice in the depolymerase treatment group survived. Compared with those in Group 4, the number of viable bacteria in the blood, lung and liver in Group 5 and Group 6 were significantly lower. In the two treatment groups, there were also significant differences in the quantity of bacteria in the blood and liver but not in the lung. Compared with those in Group 4 and Group 5, the observed indices in Group 6 significantly decreased. The mice in Groups 7, 8 and 9 survived throughout the entire process.

**Figure 8:**
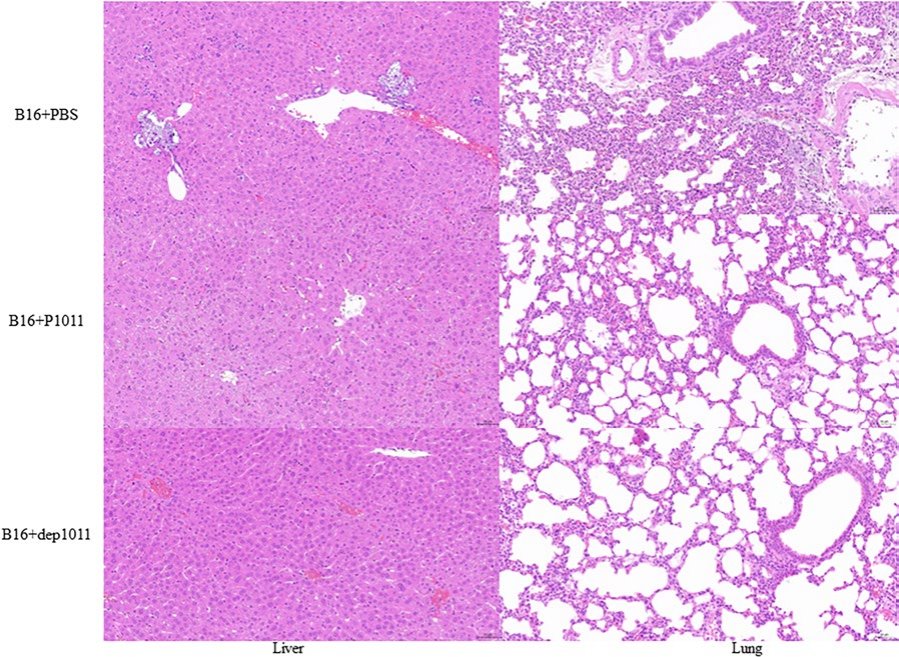
Pathological analysis of mice infected with B16 and the treatment group. H&E-stained liver and lung tissues are shown. The scale bar is 50 μm.

**Figure 9:**
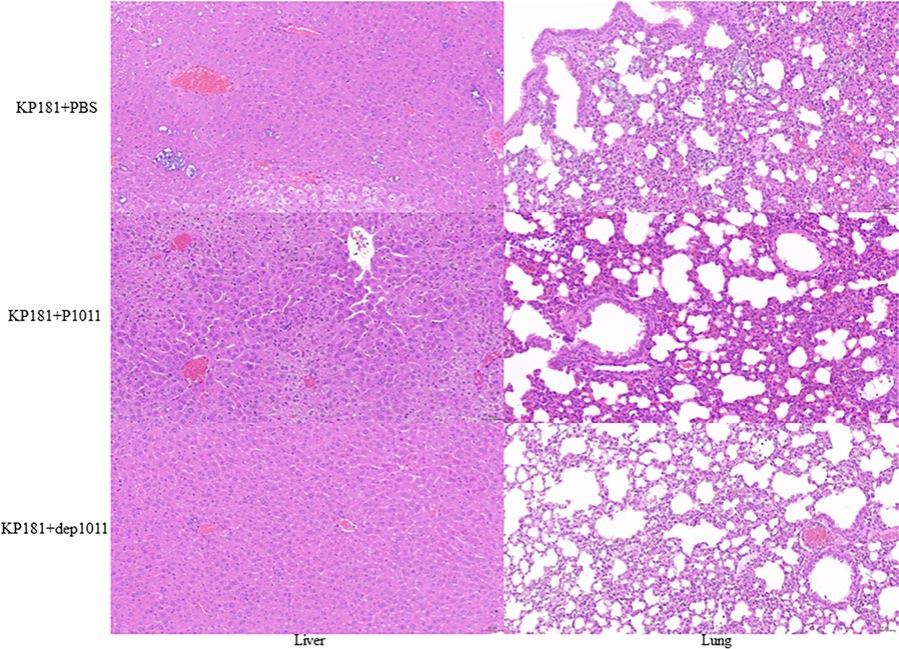
Pathological analysis of KP181-infected and treated mice. H&E-stained liver and lung tissues are shown. The scale bar is 50 μm.

Group 1 presented severe hepatocellular necrosis accompanied by hydropic degeneration along with pyknotic nuclei and nuclear fragmentation within the liver tissue sections. Lung histopathology revealed substantial granulocyte infiltration coupled with mild perivascular oedema. Group 2 exhibited minimal instances of liver cell necrosis amidst scattered ballooning degeneration. There were no obvious pathological changes in Group 3 (Figure 8). In Group 4, necrosis of liver cells, karyopyknosis and nuclear fragmentation, granulocyte infiltration, and ballooning degeneration were observed in the liver, and a large amount of granulocyte infiltration was observed in the lung. In Group 5, a small amount of necrotic and ballooning degeneration cells, karyopyknosis and nuclear fragmentation were observed in the liver tissue; a large amount of granulocyte infiltration was observed in the lung. There was no obvious pathological change in Group 6 (Figure 9).

## Discussion

*K. pneumoniae* is an encapsulated bacterium that results in a series of infections in humans and animals. Generally, antibiotic resistance among bovine *K. pneumoniae* tends to be less severe. In contrast, hospital-acquired *K. pneumoniae* strains typically exhibit multidrug resistance profiles, rendering antibiotics increasingly ineffective. In this study, we discovered that bovine *K. pneumoniae* could inherit antibiotic resistance genes from human-derived strains, consequently augmenting their own tolerance levels to antimicrobials, which is worthy of attention. Given the pressing demand for alternatives to traditional antibiotics, renewed focus has turned towards the use of phages and their derivatives as promising solutions. This study represents the first investigation into the therapeutic effects of phages and their depolymerases against K5 *K. pneumoniae* in a mouse model. This study may provide a new therapeutic agent for *K. pneumoniae* infection.

To date, nearly all hypervirulent and carbapenem-resistant *K. pneumoniae* strains are hvKP strains with carbapenem resistance plasmids (CR-hvKPs) or CRKP strains with virulence plasmids (hv-CRKPs). The latter occupy a large proportion [34]. Herein, strain B16 acquired the KPC-2 gene by conjugation, while KP181, which has a hypermucoviscous phenotype, failed under the same conditions, which agrees with previous reports that plasmids cannot be easily obtained from hvKP or that plasmids are difficult to construct [34, 35].

Phages provide promising alternatives for treating hypervirulent and carbapenem-resistant *K. pneumoniae*. With an estimated 10^31^ potential phages in existence, they represent a vast untapped resource. Our newly isolated phage P1011 differs genetically from previous K5-specific phages and possesses a unique morphology, expanding the arsenal of phages available for controlling K5 *K. pneumoniae* strains. Although great progress has been made in phage therapy, several factors limit its development [36]. One major constraint is the narrow lytic spectrum resulting from the highly specific interaction between phages and host cell receptors. Diverse bacterial structures can function as phage receptors, including capsules, outer membrane proteins, and pilus [37, 38]. Some phages utilize multiple bacterial components simultaneously as receptors [39]. For example, the coliphage Bp7 employs the LamB and OmpC proteins and the core lipopolysaccharide as receptors [40]. P1011 also infects strain B16 via multiple receptors, namely, the capsule and membrane protein OmpC (unpublished). Conversely, strain KP181 does not support P1011 propagation, probably owing to the absence of additional receptors in addition to the capsule. Another challenge facing phage therapy is phage resistance, which occurs both in vitro and in vivo [41, 42]. This may account for not all mice surviving in the phage treatment group infected with B16. We isolated and identified two different types of phage-resistant bacteria from B16, and their virulence both decreased (unpublished). Although phage resistance is widespread, it is frequently accompanied by reduced virulence and restored antibiotic sensitivity, thereby facilitating bacterial clearance.

To date, there are no reports of depolymerase resistance, which may be due to its role in degrading bacterial polysaccharides rather than directly cracking bacteria [43]. Depolymerases are generally composed of a conserved N-terminus and a variable C-terminus. BlastP analysis revealed that the N-terminus of dep1011 shares homology with the tail fibre protein (TFP) of numerous phages, while the C-terminus of dep1011 exhibits homology solely with *Klebsiella* phage K5-4 and *Klebsiella* phage K5-2. In addition, no homology was detected between the N-terminus of dep1011 and the depolymerases of *Klebsiella* phage K5-4 and K5-2. These findings suggest that the C-terminus of dep1011 may function as the enzymatic domain responsible for its depolymerase activity. To date, more than 20 types of depolymerases targeting various capsular types of *K. pneumoniae* have been identified. The development of a comprehensive depolymerase library capable of addressing all serotypes of *K. pneumoniae* could significantly alleviate the strain placed on public health resources by multidrug-resistant bacteria.

Although many studies have investigated the antibacterial properties of phages and active proteins in vivo, few studies have directly compared the therapeutic efficacy of phages and their depolymerases. We found that mice treated with depolymerase had greater survival than those treated with phage, regardless of whether the target strain supported phage proliferation. These results are in accordance with previous work showing that the antimicrobial activity of the depolymerase Dep_kpv74 was greater than that of the phage KpV74 against K2 *K. pneumoniae* [44]. However, this finding contradicts another study on the protective effectiveness of phage PHB19 and its depolymerase Dep6 against O91 Shiga toxin-producing *Escherichia coli* infection [45]. This discrepancy may be attributed to differences in the depolymerase receptor and type of bacteria. Both Dep_kpv74 and Dep1011 bind specifically to capsular polysaccharides, whereas Dep6 targets lipopolysaccharides. Capsules are critical virulence factors of *K. pneumoniae*. Capsule-stripped *K. pneumoniae* is more easily cleared by the body’s immune system [46]. The bacterial pellet of KP181 treated with dep1011 and the halo on the lawn of KP181 indicated that the capsule of KP181 could be degraded by dep1011. The free depolymerase released by P1011 may account for the substantial reduction in bacterial loads in Group 5, although the survival rate of the mice did not improve.

In conclusion, our results demonstrate that both P1011 and dep1011 exhibit significant potential as alternative therapies to traditional antibiotics. Notably, compared with P1011, dep1011 showed superior efficacy, suggesting greater suitability for medical applications. Furthermore, dep1011 offers additional benefits, such as high specificity for capsule typing and diagnostic purposes. Overall, our findings provide compelling evidence for the utility of phages and their associated enzymes as promising new tools in the fight against bacteria.

## Data Availability

The data underlying this article are available in the GenBank Nucleotide Database under accession number OR492660.
